# A rare optical coherence tomography finding of a striped, low-attenuation plaque protruding into the lumen in in-stent restenosis

**DOI:** 10.1186/s43044-025-00711-6

**Published:** 2026-01-12

**Authors:** Naoya Otaka, Hidenori Matsusaka, Kunio Morishige

**Affiliations:** Matsuyama Red Cross Hospital, Ehime, Japan

**Keywords:** In-stent restenosis, Optical coherence tomography, Drug-coated balloon, Neoatherosclerosis, Thrombus organization, Case report

## Abstract

**Background:**

In-stent restenosis (ISR) remains a clinical challenge in the drug-eluting stent (DES) era. Neoatherosclerosis is a recognized mechanism, but tissue responses after drug-coated balloon (DCB) therapy may present with atypical morphologies.

**Case presentation:**

We report a 55-year-old man with recurrent angina and ISR nine years after DES implantation and one year after DCB angioplasty. Optical coherence tomography (OCT) revealed a distinctive striped, low-attenuation plaque protruding into the lumen from outside the stent struts. This lesion lacked lipid, calcification, or macrophages, making it difficult to classify as typical neoatherosclerosis. The morphology suggested layered neointimal remodeling with possible thrombus organization after DCB treatment. The lesion was successfully treated with excimer laser coronary angioplasty, scoring balloon dilatation, and DCB angioplasty.

**Conclusion:**

This case illustrates an atypical OCT morphology observed in recurrent ISR after DCB angioplasty. Recognition of such atypical ISR morphologies may enhance understanding of neointimal healing after DCB treatment and guide interventional strategies.

**Supplementary Information:**

The online version contains supplementary material available at 10.1186/s43044-025-00711-6.

## Introduction

In-stent restenosis (ISR) after drug-eluting stent (DES) implantation remains a therapeutic challenge. Neoatherosclerosis, defined as the development of atherosclerotic changes within the neointima of DES, is recognized as one of the major mechanisms of late stent failure [1]. Optical coherence tomography (OCT) allows detailed tissue characterization and has revealed diverse ISR morphologies. Here, we describe an atypical OCT morphology characterized by a striped, low-attenuation plaque protruding into the lumen from outside the stent struts in a patient with recurrent ISR one year after drug-coated balloon (DCB) therapy.

## Case presentation

A 55-year-old man with multiple cardiovascular risk factors presented with recurrent angina. Nine years earlier, two XIENCE Alpine drug-eluting stents (Abbott Vascular, USA), sized 3.0 × 18 mm and 3.0 × 33 mm, had been implanted in the proximal and mid segments of the left anterior descending artery (LAD). One year before the current admission, a DES was implanted in the right coronary artery for chronic coronary syndrome, and DCB angioplasty was performed for ISR in the LAD. At that time, fractional flow reserve across the LAD lesion was 0.73, indicating functionally significant ischemia (Supplementary Figure A-D).

**Fig. 1:**
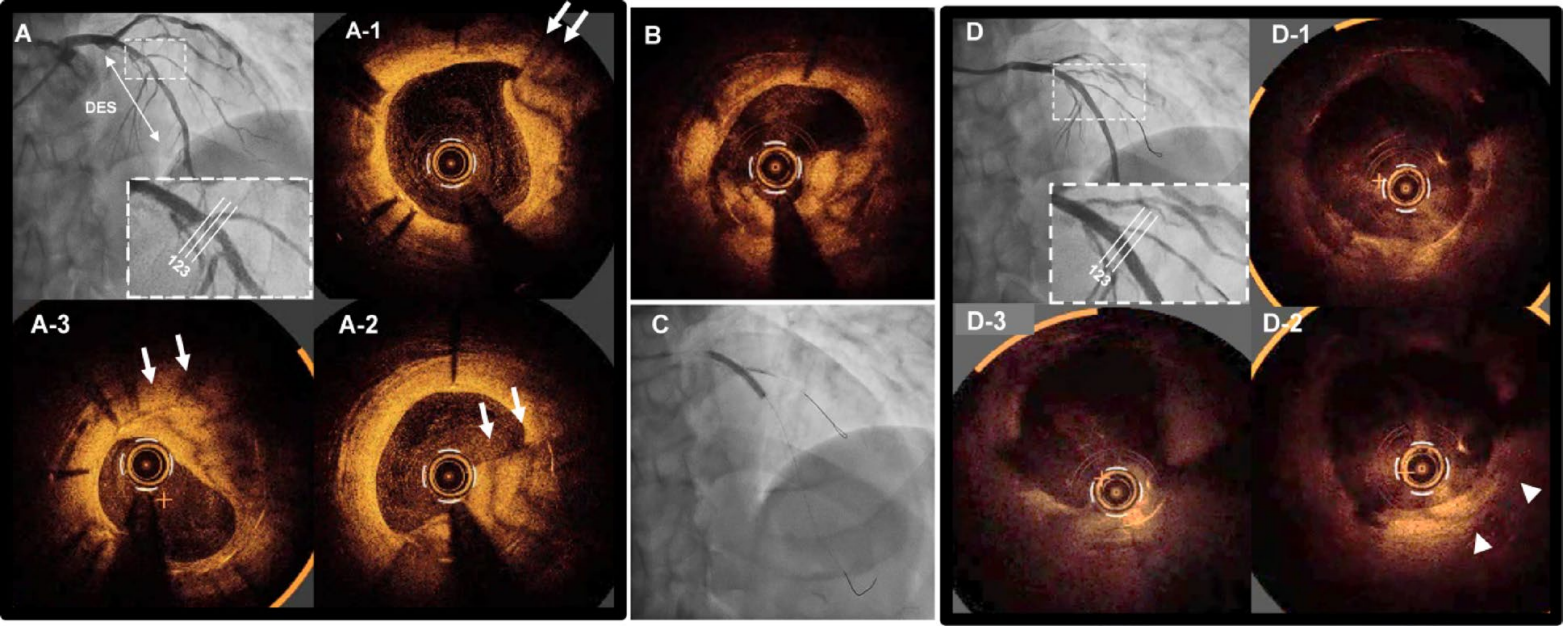
**A** Coronary angiography showing a hazy lesion in the mid-LAD. **A1–3** OCT images demonstrating a striped, low-attenuation plaque protruding into the stent lumen. Arrows indicate the characteristic striped pattern of the protruding tissue. **B** Post-excimer laser OCT showing partial reduction of the protruding plaque. **C** Final angiographic result after DCB and kissing balloon inflation. **D**, **D1–3** Final OCT images showing adequate lesion expansion and plaque compression

Follow-up angiography revealed a hazy lesion in the mid-LAD, with a fractional flow reserve of 0.72, confirming significant ischemia (Fig. 1A). PCI was therefore scheduled.

OCT (Dragonfly Opstar; Abbott Vascular) demonstrated a striped, low-attenuation plaque protruding into the lumen from outside the stent struts (Fig. 1A1–3; Supplementary Video 1). This morphology suggested layered neointimal remodeling following prior DCB therapy, possibly reflecting healed thrombus or complex healing processes, rather than classical lipid-rich neoatherosclerosis.

The lesion was treated with excimer laser coronary angioplasty (ELCA; Philips, USA; 1.4 mm C-type catheter, 60 mJ/mm², 40 Hz). Post-ablation angiography showed no apparent flow-limiting complications.

(Supplementary Video 2). Post-ablation OCT showed partial reduction of the protruding tissue (Fig. 1B). Quantitative OCT analysis demonstrated a reduction in the protruding tissue area from 4.49 mm² before excimer laser coronary angioplasty to 3.82 mm² after ELCA, suggesting effective debulking of the protruding neointimal tissue. Lesion preparation was subsequently performed using a 3.5 × 13 mm scoring balloon (Aperta NSE, Nipro, Japan). After scoring balloon dilatation, flow compromise occurred in the diagonal branch, which was managed with balloon dilatation using a Ryurei 1.5 × 10 mm semi-compliant balloon (Terumo, Japan).

Finally, a SeQuent Please Neo 3.5 × 30 mm paclitaxel-coated balloon (Nipro) was applied to the LAD, followed by snuggled kissing balloon inflation involving the diagonal branch (Fig. 1C). Final OCT demonstrated adequate lesion expansion and compression of the protruding tissue (Fig. 1D, D1–3).

## Discussion

This case highlights a distinctive OCT morphology of a striped, low-attenuation plaque protruding into the stent lumen. Unlike typical neoatherosclerosis, which is characterized by lipid deposition, calcification, or macrophage accumulation, this lesion lacked such features. Instead, its layered appearance suggested neointimal remodeling after prior DCB angioplasty, possibly reflecting thrombus healing or dissection repair.

Neointimal remodeling after DCB therapy has been increasingly recognized. A recent study reported layered tissue patterns on OCT after DCB treatment, likely representing complex healing processes [2]. Moreover, previous OCT investigations have demonstrated a variety of neointimal changes following DCB therapy, such as progression or regression of neoatherosclerosis, calcifications, uncovered struts, evaginations, and even mushroom-like protrusions [3]. These findings indicate that the present OCT appearance should be interpreted within the spectrum of known ISR morphologies, rather than as a distinct pathological entity.

This case illustrates the diverse morphological patterns of ISR and emphasizes the diagnostic value of OCT. Recognition of such atypical features may refine our understanding of vascular healing after DCB therapy and guide individualized interventional strategies.

### Limitations

This case report is limited by the absence of OCT images from the initial DCB procedure, which would have provided more detailed insights into the temporal evolution of the lesion. Although intravascular ultrasound images from the initial DCB procedure are available and presented, OCT-based tissue characterization was not performed. Furthermore, the image quality in Fig. 1D was suboptimal, although interpretation remained feasible.

## Conclusion

We report an atypical OCT morphology of a striped, low-attenuation plaque protruding into the stent lumen during recurrent ISR after DCB angioplasty. This morphology likely represents layered neointimal remodeling with thrombus healing, rather than typical neoatherosclerosis. Further case accumulation is warranted to clarify its clinical significance.

## Supplementary Information

Supplementary Material 1

Supplementary Material 2

Supplementary Material 3

## Data Availability

The authors confirm that the data supporting the finding of this study are available within the article.

## Abbreviations

DCB: Drug-coated balloon
DES: Drug-eluting stent
ELCA: Excimer laser coronary angioplasty
ISR: In-stent restenosis
LAD: Left anterior descending artery
OCT: Optical coherence tomography
PCI: Percutaneous coronary intervention
