# Supplementary figures and images for: A rare optical coherence tomography finding of a striped, low-attenuation plaque protruding into the lumen in in-stent restenosis
Source: Egypt Heart J. 2026 Jan 12;78:3. doi: 10.1186/s43044-025-00711-6 (PMC12796041; doi:10.1186/s43044-025-00711-6)

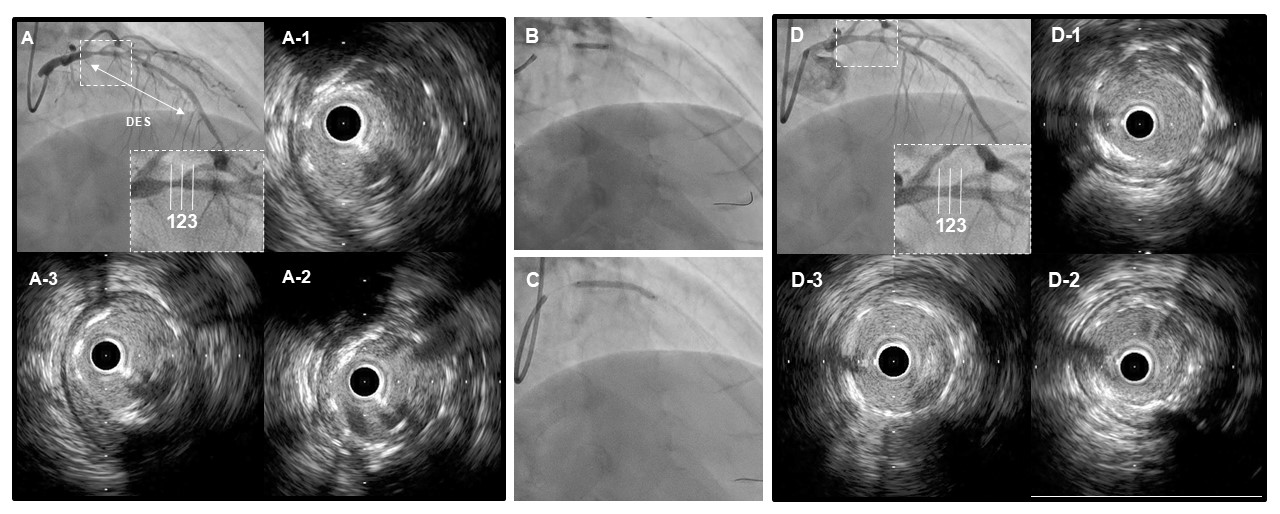

Supplement: Supplementary file 1 — Supplementary Material 1 [file 43044_2025_711_MOESM1_ESM.jpg]
